# Abilities to Explicitly and Implicitly Infer Intentions from Actions in Adults with Autism Spectrum Disorder

**DOI:** 10.1007/s10803-017-3425-5

**Published:** 2017-12-06

**Authors:** Eleanor J. Cole, Katie E. Slocombe, Nick E. Barraclough

**Affiliations:** The Department of Psychology, The University of York, Heslington, York, YO10 5DD UK

**Keywords:** Autism spectrum disorder, Intentions, Mentalizing, Autistic traits, Action perception, Eye-tracking

## Abstract

Previous research suggests that Autism Spectrum Disorder (ASD) might be associated with impairments on implicit but not explicit mentalizing tasks. However, such comparisons are made difficult by the heterogeneity of stimuli and the techniques used to measure mentalizing capabilities. We tested the abilities of 34 individuals (17 with ASD) to derive intentions from others’ actions during both explicit and implicit tasks and tracked their eye-movements. Adults with ASD displayed explicit but not implicit mentalizing deficits. Adults with ASD displayed typical fixation patterns during both implicit and explicit tasks. These results illustrate an explicit mentalizing deficit in adults with ASD, which cannot be attributed to differences in fixation patterns.

**Electronic supplementary material:**

The online version of this article (10.1007/s10803-017-3425-5) contains supplementary material, which is available to authorized users.

## Introduction

Autism Spectrum Disorder (ASD) is the term used in the most recent edition of the Diagnostic and Statistical Manual of Mental Disorders (DSM-V) to describe a range of neurodevelopmental disorders, classified according to core deficits in social communication and interaction as well as restricted and repetitive interests (American Psychiatric Association 2013). One of the social communication difficulties associated with ASD is difficulty inferring the internal states of others including their intentions, mental states and beliefs (Baron-Cohen et al. 1997; Chung et al. 2014; Frith 2001; Holt et al. 2014a, b; Jolliffe and Baron-Cohen 1999a, b), collectively referred to as mentalizing deficits. Mentalizing deficits are so strongly associated with ASD that mentalizing abilities are even assessed in diagnostic and screening tools such as the Autism Diagnostic Observation Schedule (ADOS-2) and the Autism Quotient (AQ) scale (Baron-Cohen et al. 2001; Lord et al. 2000). Due to the spectral nature of ASD, individuals without a diagnosis also display varying degrees of autistic traits. Individuals with relatively high but not clinically significant levels of autistic traits have been shown to display subtler versions of the behavioural and neurological characteristics associated with ASD (Best et al. 2015; Di Martino et al. 2009; Lindell et al. 2009; Ridley et al. 2011; van Boxtel and Lu 2013) including mentalizing deficits (Baron-Cohen et al. 2001; Chung et al. 2014; Happé 1994; Kana et al. 2014; Moran et al. 2011).

Despite the strong association between ASD and mentalizing deficits, experimental evidence regarding the nature of these deficits is inconsistent, with some studies finding that adults with ASD are impaired at inferring intentions, emotions and mental states of others (Baron-Cohen et al. 2001; Castelli et al. 2002; Happé 1994; Kana et al. 2014; Moran et al. 2011; Senju et al. 2009) and others reporting adults with ASD (Kana et al. 2009; Kirkovski et al. 2015; Ponnet et al. 2004; Roeyers et al. 2001; Spek et al. 2010) and high levels of autistic traits (Nijhof et al. 2016) show typical performances on mentalizing tasks. A number of factors may have contributed to these inconsistent findings, including whether task instructions explicitly stated that participants should mentalize, the stimuli used, the type of mentalizing assessed and the method used to measure mentalizing abilities.

The majority of previous studies have explicitly asked participants to make inferences about the internal states of others (e.g. Baron-Cohen et al. 2001; Gallagher et al. 2000; Happé 1994; Holt et al. 2014a, b; Jolliffe and Baron-Cohen 1999a, b; Kana et al. 2009, 2014; McAleer et al. 2011; Roeyers et al. 2001). Only a small number of studies have examined the capabilities of adults with ASD to infer the internal states of others when not specifically told to do so; this is known as ‘implicit mentalizing’. The existing adult literature shows consistent implicit mentalizing deficits associated with ASD (Castelli et al. 2002; Rosenblau et al. 2015; Schuwerk et al. 2014; Senju et al. 2009) but the explicit mentalizing data are inconsistent (Baron-Cohen et al. 1997; Castelli et al. 2002; Kana et al. 2009; Kirkovski et al. 2015; Ponnet et al. 2004). It may be that the instructions given concerning which elements should be attended to during explicit tasks, allow some high functioning adults with ASD to perform at a typical level, which they would be unable to do without the explicit instructions.

The apparent existence of a consistent implicit mentalizing deficit but lack of a consistent explicit mentalizing deficit in adults with ASD in the existing literature may, however, be attributable to other confounding factors, including stimuli differences. Most studies that have reported implicit mentalizing deficits in adults with ASD have used movie stimuli (e.g. Rosenblau et al. 2015; Schuwerk et al. 2014; Senju et al. 2009), which were more complex and naturalistic than stimuli used in the majority of explicit tasks. The stimuli used in the majority of explicit mentalizing tasks were passages of text, still images or cartoon strips which provide very simplistic representations of social interactions and a number of these studies found no mentalizing deficits in adults with ASD (e.g. Kana et al. 2009; Kirkovski et al. 2015; Ponnet et al. 2004; Roeyers et al. 2001; Spek et al. 2010). In support of this argument, two previous studies (Ponnet et al. 2004; Roeyers et al. 2001) investigated the abilities of adults with Pervasive Development Disorders (PDD; including ASD) to explicitly infer the mental states of others using both simple stimuli (images of people’s eyes and short passages of text) and naturalistic videos of social interactions. The adults with PDD were not impaired on the explicit mentalizing tasks that used the simple stimuli but did show impairments with the more complex naturalistic stimuli (Ponnet et al. 2004; Roeyers et al. 2001). Additionally, the only previous study that has investigated both implicit and explicit mentalizing abilities using complex, naturalistic stimuli found that adults with ASD displayed equivalent impairments on both implicit and explicit tasks (Rosenblau et al. 2015).

Differences in the way mentalizing performances have been measured may have also contributed to existence of consistent implicit mentalizing deficits but inconsistent data regarding explicit mentalizing abilities in the previous adult literature. Some studies have measured implicit mentalizing abilities using eye-tracking data alone (Schuwerk et al. 2014; Senju et al. 2009). In these studies, participants watched animations in which a character wrongly believed an object was in a certain location. Adults with ASD spent shorter periods fixating on the place in which the character wrongly believed the object was located. This was interpreted as impaired implicit mentalizing. However, a number of studies have reported that adults with ASD have unusual patterns of eye gaze when processing social stimuli (Kliemann et al. 2010; Pelphrey et al. 2002) and unusual fixation patterns have been found during face processing tasks in the absence of behavioural differences (Rutherford and Towns 2008; Spezio et al. 2007). Therefore, adults with ASD may be able to deduce the internal states of others despite atypical eye movements. In contrast, explicit mentalizing studies have always used measurable behavioural outcomes to assess mentalizing abilities.

The term ‘mentalizing’ covers a variety of internal state inferences which may involve different processes (Call and Tomasello 2008; Pineda and Hecht 2009); it is possible that the different internal state inferences required across studies may have also contributed to the heterogeneity in the literature. Previous studies have reliably found that adults with ASD are impaired at inferring others’ intentions (Kana et al. 2014; Murdaugh et al. 2014; Ponnet et al. 2004; Roeyers et al. 2001) and others’ emotions (Atkinson 2009; Cassidy et al. 2013; Enticott et al. 2013; Hubert et al. 2007; Nackaerts et al. 2012). However, the existing literature is more inconsistent regarding abilities to infer others’ mental states (Baron-Cohen et al. 1997; Kana et al. 2009; Kirkovski et al. 2015; Kleinman et al. 2001; Roeyers et al. 2001; Spek et al. 2010) or false beliefs (Frith and Happé 1994; Schuwerk et al. 2014; Senju et al. 2009). The neuroimaging and developmental literature also support the argument that the different subcomponents of mentalizing reflect different processes; the results of a meta-analysis suggest that children develop the ability to infer others’ desires before they are able to infer others’ beliefs and can detect others’ emotions before they can deduce false beliefs (Wellman and Liu 2004). Additionally, neuroimaging studies have shown that different brain areas are active during mentalizing tasks depending on the inferences being made (Pineda and Hecht 2009; Schurz et al. 2014). Collectively, these data suggest that the subcomponents of mentalizing are distinct processes associated with different brain areas and developmental trajectories.

In summary, although ASD is associated with mentalizing deficits, the nature of these deficits is unclear. The existing literature suggests that adults with ASD are more likely to show impaired performances on implicit mentalizing tasks using complex naturalistic stimuli that probe understanding of intentions or emotions. To our knowledge, only one study to date has assessed both implicit and explicit mentalizing abilities in adults with ASD using measurable behavioural outcomes (Rosenblau et al. 2015). In this study, a comparison between adults with and without ASD found that participants with ASD showed reduced abilities to both implicitly and explicitly infer the mental states of actors from short movies but there was no difference in the degree of impairment between tasks. However, this study did not use a non-mentalizing control task so it is unclear whether the poorer performances observed in adults with ASD were specifically due to mentalizing deficits or whether poorer performances reflect reduced abilities to perform the experimental tasks in general. Thus the current study aimed to test the abilities of adults with ASD to both implicitly and explicitly mentalize, using complex stimuli, measurable behavioural outcomes and a non-mentalizing control task.

This study specifically investigated the abilities of adults with ASD to both implicitly and explicitly infer the intentions of others from the kinematics of their hand actions using the same naturalistic stimuli. Previous studies have shown that hand actions with different intentions display subtle differences in action kinematics and adults without ASD are able to infer others’ intentions from these differences in action kinematics (Ansuini et al. 2015; Sartori et al. 2009). In the first experiment, participants watched videos of actors playing a poker chip game and had to decide which actor, from a choice of two, they would prefer to play the poker chip game with. Participants were shown one video depicting an actor deliberately not passing a poker chip to another player (‘spiteful’ action) and a video of another actor accidentally not passing a poker chip to another player (‘clumsy’ action). In this task, participants were not explicitly asked to infer actors’ intentions; rather participants’ choice of actor was dependent upon ‘covert’ mentalizing (implicit mentalizing task). In contrast, during the second experiment, participants watched the same movies and were explicitly asked to infer the intentions of the actors. In addition to contrasting the performance of the ASD and typically developing groups, due to the spectral nature of ASD, we then examined the relationship between the level of autistic traits displayed and abilities to infer others’ intentions across all participants. We also tracked participants’ eye movements during both experiments in order to test whether any potential behavioural differences associated with autistic traits could be explained by atypical fixation patterns (cf. Schuwerk et al. 2014; Senju et al. 2009). It was predicted that adults with ASD would display reduced abilities to infer the intentions of others compared to matched control participants and across all participants higher levels of autistic traits would predict poorer performances. We also hypothesised that mentalizing deficits associated with ASD would be more evident in the implicit task compared to the explicit task.

## Methods

### Participants

Twenty-one adults with Autism Spectrum Disorder (ASD; 14 male) were recruited for this study. The majority of the ASD sample were students from the University of York (n = 13) and the remaining ASD participants were recruited from a local support group. Four participants were excluded for having scores that were not significantly higher than chance on the control task (see below). This resulted in a final participant sample of 17 adults with ASD (10 male ages 18–56, mean age = 23.71, SD = 9.24) and 17 individually age, sex and IQ matched control participants (TD—Typically Developing; ages 18–55, mean age = 23.71, SD = 9.07). See Table 1 for participant demographics.

**Table 1:** Participant demographic information; group mean (SD) values

	ASD	TD	p
Age	23.71 (9.24)	23.71 (9.07)	0.97
Gender (male:female)	10:7	10:7	1.00 (X^2^)
IQ (WASI)^a^	120.12 (9.32)	120.00 (10.09)	0.93
WASI verbal score^b^	62.88 (6.66)	61.61 (7.52)	0.86

p values were derived from a one-way MANOVA unless otherwise stated

^a^The IQ scores were obtained using the two-subtest version of Wechsler Abbreviated Scale of Intelligence (WASI)

^b^The verbal WASI scores given are standardised scores (T-scores)

All participants in the ASD group had a clinical diagnosis of Asperger’s (n = 14) or Autism Spectrum Disorder. All diagnoses were issued by qualified clinicians external to this study. None of the ASD participants had a history of delayed language development or existing learning difficulties. All participants had IQ scores above 100. All neurotypical participants reported that they had no neurological disorders and adults diagnosed with ASD reported no other neurological conditions.

Experiments were approved by the ethics committee of the Department of Psychology, University of York, and were performed in accordance with the ethical standards outlined in the 1990 Declaration of Helsinki.

### Psychological Tests

The Autism Diagnostic Observation Schedule (ADOS-2; Lord et al. 2000), Social Responsiveness Scale (SRS; Constantino et al. 2003), The Awareness of Social Inference Test (TASIT; McDonald et al. 2006), Autism Quotient (AQ; Baron-Cohen et al. 2001) and Wechsler Abbreviated Scale of Intelligence (WASI; Wechsler 1999) were administered to all participants. The ADOS-2 assessments were filmed and then scored by both the experimenter and an independent rater who was blind as to whether participants had a diagnosis or not. Both the experimenter and independent rater were trained to the level of research reliability on the ADOS-2 assessment. If the ADOS-2 scores differed between the experimenter and independent rater, the assessment movies were re-watched and a final score was agreed on. The independent ADOS-2 scores never differed by more than 2 points between the raters. The SRS and TASIT are designed to detect social impairment. The SRS is a self-report measure and TASIT measures abilities to detect sarcasm and lies from movies showing social interactions. The AQ is a self-report measure of autistic traits. The two subtest version of the WASI was used to measure the IQ of participants. All these psychological tests have been shown to have good psychometric properties (Allison et al. 2011; Constantino et al. 2003; Hurst et al. 2007; McDonald et al. 2006; Oosterling et al. 2010).

### Stimuli

**Fig. 1:**
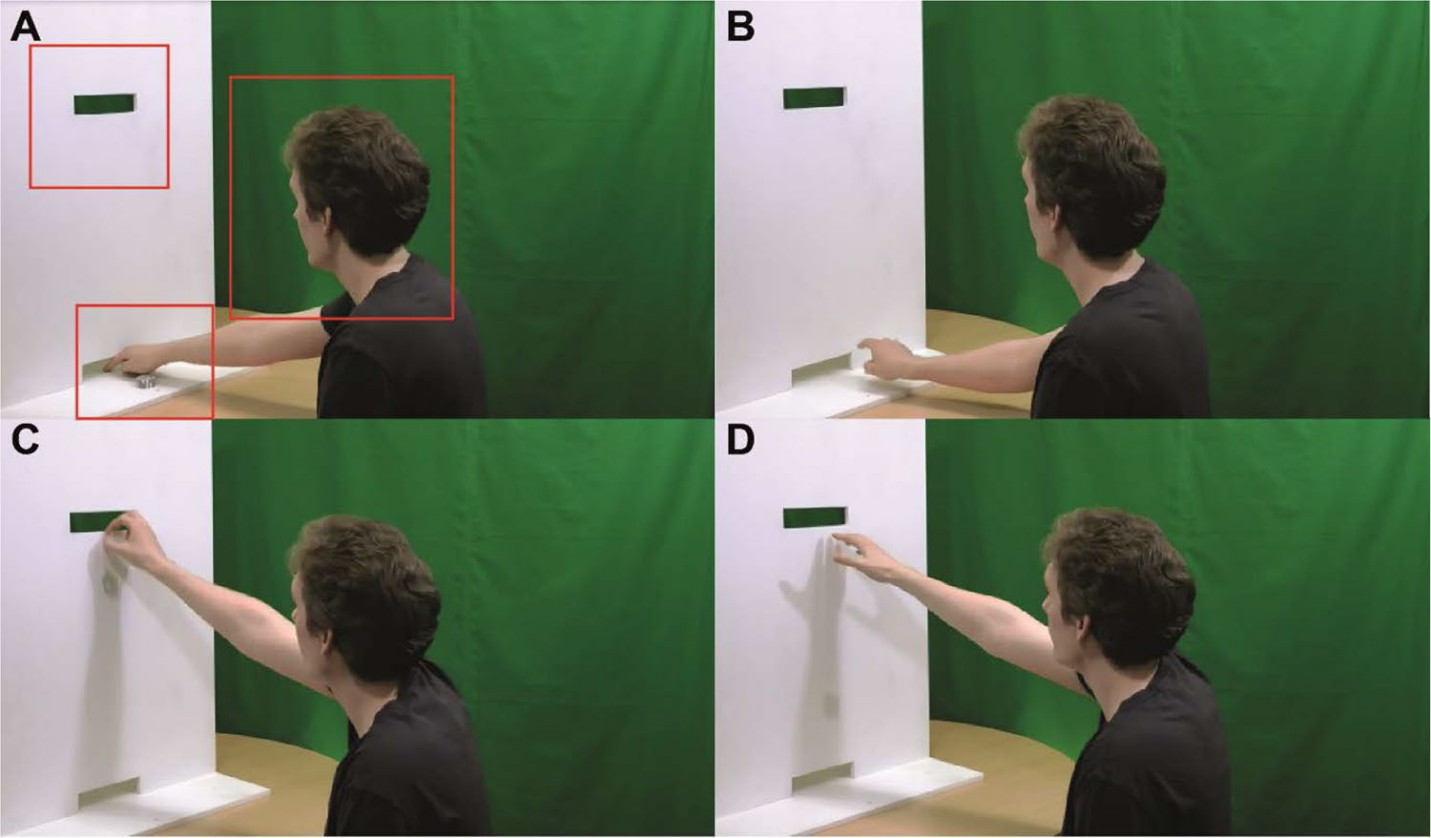
Example screenshots from the hand action movies depicting positive and negative intentions. **a** The actor pushes five poker chips with a positive intention (clumsy 5 pushing action). **b** The actor deliberately pushes a poker chip away from the slot (spiteful 1 pushing action). **c** The actor accidentally drops a poker chip (clumsy 1 grasping action). **d** The actor deliberately drops the poker chip (spiteful 1 grasping action). The squares overlaid onto action **a** illustrate the regions of interest (ROIs) used for the eye-tracking analyses

The movie stimuli were designed to show different actors playing a poker chip exchange game. The poker chip game involved passing poker chips to another player through slots in a white wooden board (see Fig. 1). Ten different types of hand actions were filmed (Panasonic TM900 HD-DV camera; 1920 × 1080 pixels at 50 Hz progressive scan). Five of the hand actions involved pushing poker chips with the index finger of the right hand through a slot in the board which was level with the surface of the table. The other five hand actions involved grasping poker chips with the index finger and thumb of the right hand and passing them through a slot in the board at head height. Two different types of actions were used to generalise results across different action types. Both pushing and grasping actions were executed by the actor in five different ways: (1) clumsily failing to pass one poker chip—here the actor had a positive intention to pass the chip to the other player, but the outcome of the action was unsuccessful (clumsy 1); (2) Clumsily failing to pass five pokers chips; positive intention to pass the chips, but the outcome of the action was unsuccessful (clumsy 5); (3) Spitefully (deliberately) failing to pass one poker chip; no intention to pass the chip to the other player and the outcome of the action was unsuccessful (spiteful 1); (4) Successfully passing one poker chip; the actor intended to pass the poker chip and the action was successful (successful 1); (5) Successfully passing five poker chips; the actor intended to pass the poker chips and the action was successful (successful 5).

Twenty-eight different actors (14 female) were filmed performing all ten actions, from a three-quarters view from behind at an angle that allowed their right hand to be seen in front of them for the entire duration of the hand action but only showed a limited side profile of their face. This prevented participants from using facial information to infer the intentions of actors and required intentions to be inferred from the action kinematics alone (cf. Sartori et al. 2009; Ansuini et al. 2015). This was done in order to investigate whether adults with ASD are impaired at inferring others’ intentions irrespective of reduced fixation on the eyes, which has been well reported (Bird et al. 2011; Kliemann et al. 2010; Papagiannopoulou et al. 2014; Tottenham et al. 2014).

The actors sat in front of a white wooden board measuring 84 × 61 cm with two slots (4 × 17 cm) cut out of it (see Fig. 1). Actions started with the actor’s right hand resting on a small marker for 3 s. In order to ensure all hand actions lasted approximately 2 s, a buzzer indicated to the actors when to move their hand towards the poker chips and signalled again to indicate when the actors should let go of the poker chips. Actors performed each of the 10 different actions at least 3 times; for each actor the action with the best timing and that best depicted the particular intention was selected for the final movie. Movies were edited (Sony Vegas Pro 10) to finish 0.4 s after the poker chips left the actors’ hands; for grasping actions, this was always before the poker chips hit the table. In addition, the starts of all movies were trimmed such that they lasted exactly 4 s. Editing the movies in this way meant that movement onset occurred at slightly different times in each movie (frames 32–146).

The chosen movies were rated by 30 independent observers who were students at the University of York. Observers classified each action as either ‘clumsy’, ‘spiteful’ or ‘neither’ by pressing one of three keys on the computer keyboard. Clumsy responses were coded as − 1, spiteful responses were coded as 1 and neither responses were coded as 0. For each action, scores were averaged across participants to generate an index of the degree of ‘spitefulness’ conveyed by each movie where − 1 indicates a strong evaluation of the action as clumsy, + 1 indicates a strong evaluation of the action as spiteful, and 0 indicates an evaluation of the action as neither clumsy or spiteful. Spiteful videos were required to have spitefulness indexes higher than 0.4 and clumsy videos were required to have indexes below − 0.4 to be included in the stimuli set. Three clumsy movies had spitefulness indexes that were higher than − 0.4 and therefore were deemed to not clearly portray the desired intention (0.16, 0.03 and − 0.03 spitefulness indexes). These movies were replaced with new stimuli which were rated by another 30 independent observers and these stimuli all obtained ratings lower than − 0.4. The final stimuli used fell into three significantly (F(2,165) = 1644.94, p < .001, η_p_^2^ = .95) distinct groups; clumsy (M = − .68, SD = 0.15), spiteful (M = 0.80, SD = 0.13) and successful (M = 0.01, SD = 0.03) actions.

### Experiment 1 (Implicit mentalizing): Design and Procedure

Experiment 1 tested the participants’ abilities to implicitly infer the intentions of others from their hand actions. The task was adapted from one previously used with children (Behne and Carpenter 2005) and chimpanzees (Call et al. 2004). In these studies, experimenters either deliberately or ‘accidentally’ did not give the chimpanzees or children rewards (in the form of food or a toy respectively). Both the chimpanzees and the children attempted to interact with the experimenters for longer when experimenters accidentally dropped the reward rather than when they deliberately did not give the reward. This implied the experimenters’ intentions had been implicitly inferred and consequently the appropriate social decisions were made.

In our experiment, each participant took part in a poker chip exchange game with the experimenter prior to the main experiment in order to familiarise them with the actions shown during the experiment, and to demonstrate the value of receiving poker chips from a partner. Participants were told that the experimenter would start with 8 poker chips that were each worth one pound. However, in order for the experimenter to receive money for their poker chips at the end of the game, they had to give at least one poker chip to the participant. If the experimenter had all the poker chips on their side of the board at the end of the game, neither the experimenter nor the participant would receive any money. The experimenter then had three chances to make a deal with the participant; they would pass some poker chips through the slots in the wooden board to the participant on the other side. The participant had to accept or reject the number of poker chips that were offered each turn. If the participant accepted then they would receive a pound coin for every chip on their side, if they rejected the number of poker chips offered, then the experimenter would have to offer a different number of chips. If no agreement was reached after three rounds then neither the participant nor the experimenter received any money. The aim of the game for the participant was to end up with as many chips as possible on their side of the board. Every participant played the poker chip game four times to gain a good understanding of the purpose of passing the chips and the value of the chips (three times as the participant and once in the experimenter role). Over the three games in the participant role, each participant experienced (i) a round in which the experimenter acted spitefully (experimenter offered no chips to the participant and explained they were doing so in order to reduce the number of chances to make a deal and increase the chances of the participant accepting a lower offer); and (ii) a round in which the experimenter acted clumsily (experimenter accidentally dropped the poker chips and thus failed to make an offer) so that all participants had practical experience of both clumsy and spiteful actions. Participants also played one game in which they switched roles with the experimenter to ensure they understood the game fully.

A PC running MATLAB R2015a controlled the experiment and recorded participant responses. Participants sat approximately 60 cm from an Acer GD245HQ 24″ HD monitor on which all stimuli were presented. Participants’ eye movements were recorded during the experiment using an EyeTribe eye tracker (The EyeTribe Abs, Copenhagen). Participants rested their heads in a chin rest and fixation data from both eyes was recorded at 30 Hz. A 9-point calibration procedure was carried out before conducting each experiment. Participants for which the eye-tracker could not reach a satisfactory level of accuracy on the calibration (3/5 star rating; indicating < 1° accuracy) were excluded from subsequent eye-tracking analysis. Eye tracker data recording was controlled using the EyeTribe MATLAB toolbox (Dalmaijer; available on GitHub: https://github.com/esdalmaijer/EyeTribe-Toolbox-for-Matlab).

**Fig. 2:**
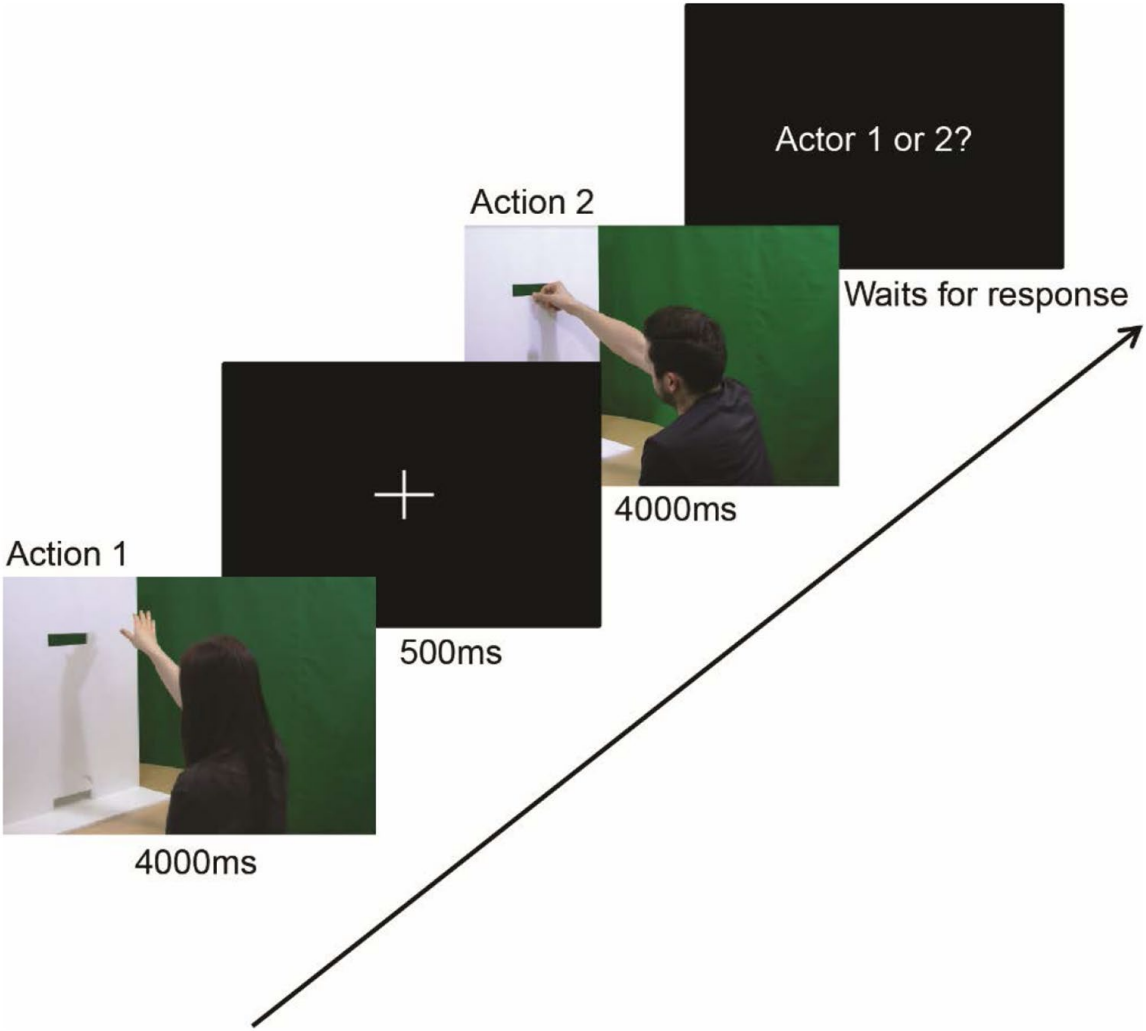
Sequence of stages during a Mentalizing trial in the implicit task. Action 1 shows a female actor deliberately dropping a poker chip (spiteful 1) and action 2 shows a male actor accidentally dropping a poker chip (clumsy 1). In order to decide whether to interact with actor 1 or actor 2 the participant must infer the intentions of the actors from the kinematics of their actions because the outcomes of the two actions are identical

Participants were told that they would watch movies of individuals playing the poker chip game they had just played themselves. Each movie would show a player’s first attempt to offer poker chips to someone on the other side of the board. The participants watched pairs of movies and had to decide subsequently whether they would rather continue playing the poker chip game with the actor in the first or the second movie. Each trial consisted of two actions of the same type (either both grasping or both pushing) presented sequentially with an inter-stimulus interval (ISI) of 1000 ms, during which the screen was black except for a white fixation cross. Following the second movie a response screen was displayed and participants had to indicate whether they would rather interact with the actor in the first or second movie by pressing either 1 or 2 on the keyboard (see Fig. 2).

Different forms of decision making were required to make a choice between the first and second actors in three different conditions; we refer to them as ‘Mentalizing’, ‘Action’ or ‘Either’ conditions. (1) Mentalizing condition: correct decisions could be based upon inferences of intention only and consisted of movies of an action with a positive intention (clumsy 1) and an action with a negative intention (spiteful 1). Here, in order to decide between the actors, participants needed to infer the intentions of the actors from the kinematics of their actions. The outcomes of the actions were the same (both actors failed to pass a poker chip to another player) but the intentions of the actors were different. (2) Action condition: correct decisions could be based upon action recognition only, consisted of movies of actors successfully passing poker chips (successful 1 and successful 5). Here, in order to decide between the actors, participants needed to recognise whether the actor was passing 1 or 5 poker chips, and did not require participants to mentalize in order to complete the task. (3) Either condition: decisions were based upon recognition of the action, or possibly inferences of intention, and consisted of movies of actors attempting to pass poke chips (clumsy 1 and clumsy 5). Here, in order to decide between the actors, participants were expected to focus on the number of chips being offered and choose the actor trying to pass the higher number of poker chips, but participants may have automatically processed the actors’ intentions and recognized that both actors have the same positive intention. This condition was included in order to test for the differences in success of the actions between the Mentalizing and Action conditions, given that Mentalizing trials always showed unsuccessful actions and Action trials always showed successful actions; Either trials always showed unsuccessful actions but did not require mentalizing.

At the start of testing, participants completed six practice trials (two of each condition) in order to familiarise them with the experimental procedure. The stimuli used in the practice trials were not included in the main experiments and the actors compared against each other in the implicit practice trials were not compared against each other in the main implicit experiment.

Participants completed 72 trials in total, viewing 144 actions (12 repeats of each action except clumsy 1 which was shown 24 times); trials lasted approximately 12 s depending on response times, and testing took approximately 15 min. The same actor never performed the same action (e.g. spiteful1 pushing action) twice, such that participants did not learn to associate certain behaviours with specific individuals. Every actor was seen the same number of times and each actor performed a preferable action 50% of the time; actor gender was also counterbalanced. Condition order was randomised and action order was counterbalanced so that the preferred action would occur first in 50% of the trials, e.g. on 50% of the Mentalizing trials the clumsy movies were shown before spiteful movies.

### Experiment 2 (Explicit mentalizing): Design and Procedure

In the second experiment, participants were asked explicitly to report the intentions of actors presented in movies. Participants returned approximately 3 months (average 112 days) after they completed Experiment 1 to complete Experiment 2. This helped minimalize the possibility of participants’ previous implicit judgements influencing their explicit judgements of the actions. Two of the ASD participants were unable to return to complete the explicit experiment, leaving a sample of thirty participants (15 matched pairs) in the explicit experiment.

As with experiment 1, participants first completed six practice trials (two of each stimulus type), in order to familiarise them with the experimental procedure. Participants then viewed all 144 of the movies seen in the Experiment 1. After each movie, participants had to indicate whether they thought the movie showed a ‘spiteful’ (deliberate), clumsy (accidental) or successful action by pressing 1, 2 or 3 respectively on the computer keyboard. The experiment consisted of two blocks of 10 min (72 movies shown in each). Each block contained 36 clumsy actions, 12 spiteful actions and 24 successful actions, the order of movies was randomised within each block and no movies were repeated. A response screen was shown after each movie until the participant responded. The PC, display and eye-tracker were all identical to Experiment 1.

### Behavioural Performance Analysis

For Experiment 1, the numbers of correct responses each participant gave in each condition (Mentalizing, Either, Action) were calculated. All 34 participants included in the analyses had scores significantly higher than chance in the Action condition (Binomial test (0.5), p < .05, scores > 17/24), indicating that all individuals understood the task. We then subtracted the number of correct responses on the Action condition from the number of correct responses on both the Mentalizing and Either condition for each participant. This allowed us to identify any task specific deficits rather than generalised poorer performances on experimental tasks.

For Experiment 2, we calculated the proportion of correct responses for the mentalizing conditions (clumsy and spiteful actions) and non-mentalizing condition (successful actions) for each participant. Similar to Experiment 1, differences between mentalizing and non-mentalizing conditions were calculated to provide a specific measure of the ability of participants to explicitly infer the intentions of others, whilst controlling for ability to do a simple action discrimination task.

Task-specific scores were not normally distributed even after log transformations had been applied. Therefore, non-parametric analyses (Mann–Whitney U tests) were used to investigate group differences in mentalizing abilities. Further, due to the spectral nature of ASD, linear regressions were used to examine the influence of autistic traits (continuous independent variable) on task-specific performances (continuous dependent variables). These linear regressions were conducted in order to identify whether any significant group differences that were found also showed a significant relationship with the continuum of autistic traits across all participants. In order to obtain a single score for each participant that reflected the level of autistic traits that they displayed, we performed a principal components analysis (PCA) on all the psychological test scores (ADOS-2, AQ, SRS and TASIT). The only factor with an eigenvalue higher than Kaiser’s criteria of 1 was extracted and used as a measure of autistic traits. Data analysis was carried out using R i386 3.2.3 (The R Foundation for Statistical Computing, Vienna, Austria, http://www.r-project.org).

### Eye-Tracking Analysis

Eye tracking data was analysed using the EyeMMV MATLAB toolbox (Krassanakis et al. 2014). Data from the implicit and explicit experiments were analysed in the same way. First, heatmaps were created using the data from all participants in order to identify regions of interest (ROIs); these were: the head of the actor, the initial start position of the hand with the poker chips, and the grasp release point. Three rectangular ROIs were drawn for each movie outlining these areas of interest. Due to the similarity in the spatial extent of the actions on the screen it was then possible to combine the co-ordinates of the ROIs from all 144 movies to make a single set of ROIs that encompassed the ROIs from all movies (see Fig. 1a). We then calculated the number and duration of fixations within each ROI during each condition for each participant. We defined the minimum duration for fixation detection as 150 ms.

The duration of fixations in each ROI as a percentage of the total number of fixations were calculated for each participant in each condition. As for the behavioural data, for Experiment 1 the duration each participant fixated in each ROI during the Action condition was subtracted from the time spent fixating in each ROI during the Mentalizing and Either conditions. For Experiment 2, the durations of fixation in each ROI during the non-mentalizing condition were subtracted from the durations of the fixation in each ROI during the Mentalizing condition. For Experiment 1, group differences in fixation patterns were tested using separate mixed-model ANOVAs for each ROI (with condition [Mentalizing-Action, Either-Action] as the within subjects variable and diagnosis as the between subjects variable). For Experiment 2, the eye-tracking data were found to violate the assumption of normality even after a log transformation had been applied so non-parametric Mann–Whitney U tests were conducted to examine potential group differences in mentalizing-specific fixation patterns. For both experiments, linear regressions were used to examine the influence of autistic traits on changes in the duration of fixations in each ROI across conditions. The data from different ROIs were treated separately because the data were not independent (participants could only fixate in one ROI at a time).

## Results

### Psychological Tests

All psychological assessment scores were highly correlated with each other except for IQ which did not correlate with the scores on any other psychological tests (Bivariate Pearson correlations; see Table 2). Three female participants with an ASD diagnosis obtained ADOS scores below the clinical cut-off. However, all of these participants had AQ scores above the clinical cut-off as well as SRS scores that indicated either moderate or severe social impairments (see Table 3 for group means scores on all psychological assessments).

**Table 2:** Correlations between psychological test scores

	1	2	3	4
1. ADOS				
2. AQ	.74***			
3. SRS	.77***	.90***		
4. TASIT	.54***	.73***	.76***	
5. IQ	.04	.17	.09	.10

***p < .*001*

**Table 3:** Participants’ psychological test scores; group mean (SD) values

	ASD	TD	p	η_p_^2^
ADOS	8.47 (2.58)	2.76 (1.86)	< .001	.63
AQ	35.71 (6.47)	16.47 (6.57)	< .001	.70
TASIT	49.24 (8.61)	57.76 (3.72)	.001	.31
SRS	114.12 (24.26)	42.76 (18.87)	< .001	.74
Autistic traits	.84 (.63)	− .84 (.42)	< .001	.73

p values were obtained from one-way MANOVA

Given that the psychological test scores assessing autistic traits were highly correlated with each other (all rs > 0.54) they were suitable for principal component analysis, the Kasier-Meyer-Olkin measure of sampling accuracy was 0.81 (above 0.6) and Barlett’s test of sphericity was significant χ^2^(6) = 108.82 p < .001. Additionally, the communalities were all above 0.7 supporting the inclusion of all the psychological tests in the principle components analysis (PCA). PCA with varimax rotation was used. The initial eigenvalues from the PCA analysis showed that one factor (with an eigenvalue of 3.23) explained 80.81% of the variance in psychological test scores. No other factors had eigenvalues higher than Kaiser’s criteria of 1 and therefore only one factor was extracted. This factor was labelled ‘autistic traits’ (see Table 3 for group mean values).

### Experiment 1

**Fig. 3:**
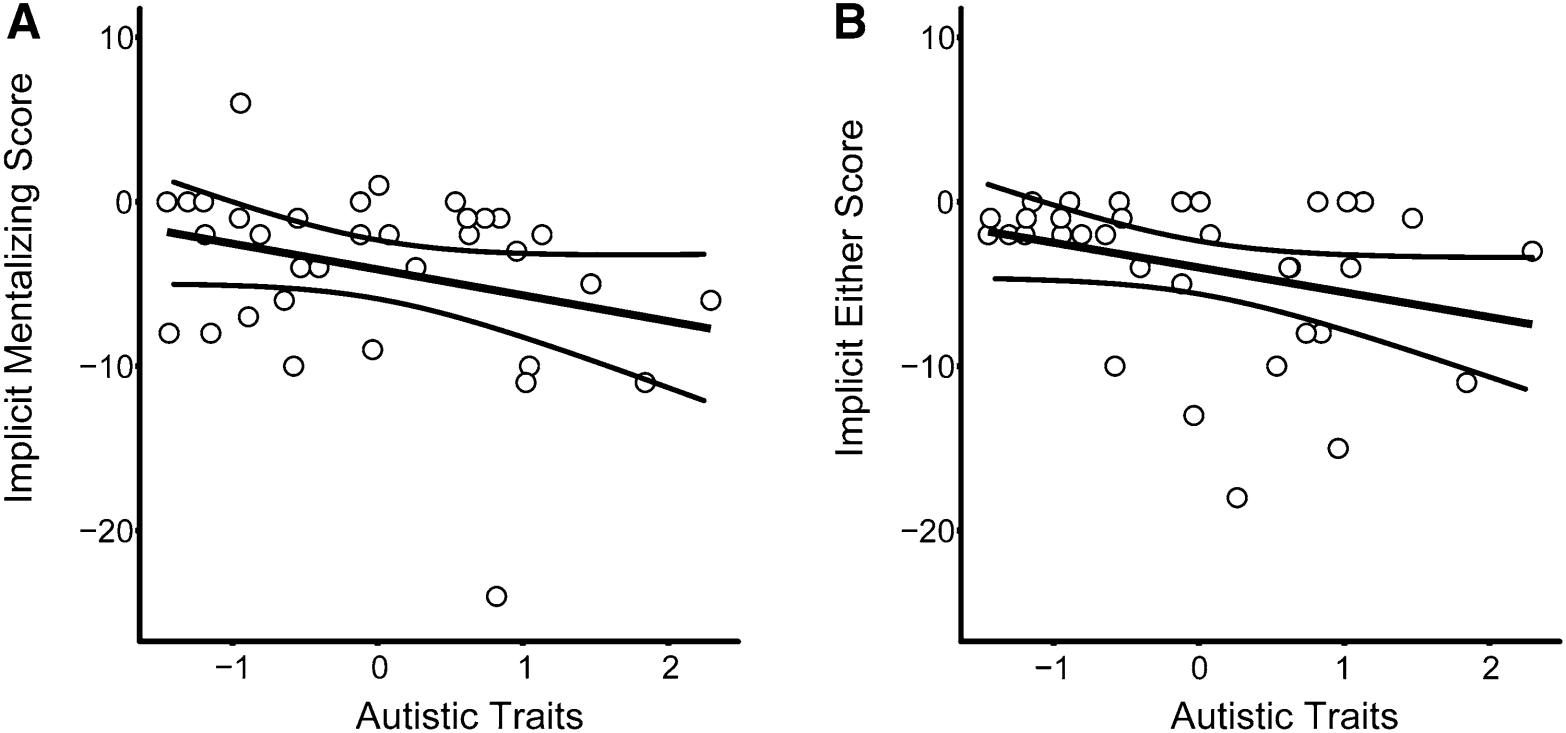
The relationship between the levels of autistic traits displayed and performances on the implicit task in the Mentalizing condition (**a**) and Either condition (**b**). Although there was a trend of poorer performances with high levels of autistic traits, linear regression analysis found that the level of autistic traits displayed was not a significant predictor of performance in the Mentalizing (F(1,32) = 3.11, p = .09, R^2^ = 0.09) or the Either condition (F(1,32) = 3.54, p = .07, R^2^ = 0.10). The curved lines represent 95% confidence intervals

ASD participants displayed poorer performances on the implicit task than matched controls (see Table 4) but group differences were not significant (Mentalizing-Action scores: U = 112.50, p = .27, r = .19; Either-Action scores: U = 90.00, p = .06, r = .33). Linear regression analyses also showed that higher levels of autistic traits were associated with poorer performances on the implicit task but this trend was not significant (see Fig. 3; Mentalizing-Action scores: F(1,32) = 3.11, p = .09, R^2^ = 0.09, 95% CI [− 5.91, − 2.33]; Either-Action scores: F(1,32) = 3.54, p = .07, R^2^ = 0.10, 95% CI [− 3.14, 0.124]).

**Table 4:** Group behavioural performances; median (IQR) values

	ASD	TD
Implicit mentalizing-action	− 3.00 (6.50)	− 2.00 (6.50)
Implicit either-action	− 4.00 (10.00)	− 2.00 (1.50)
Explicit mentalizing-non-mentalizing	− 1.88 (3.50)	− 0.74 (1.13)

In addition, adults with ASD did not show atypical changes in fixation patterns between conditions in the implicit experiment and changes in fixation patterns were not significantly different across Mentalizing and Either conditions for any of the ROIs [head ROI: task [F(1,26) = 0.45, p = .51, η_p_^2^ = .02], diagnosis [F(1,26) = 0.77, p = .39, η_p_^2^ = .03], task*diagnosis interaction [F(1,26) = 0.23, p = .63, η_p_^2^ = .01; Poker chip ROI: task [F(1,26) = 2.41, p = .13, η_p_^2^ = .09], diagnosis [F(1,26) = .32, p = .57, η_p_^2^ = .01], task*diagnosis interaction [F(1,26) = 0.70, p = .41, η_p_^2^ = 0.03]; Release point ROI: task [F(1,26) = 3.27, p = .08, η_p_^2^ = .11], diagnosis [F(1,26) = 2.99, p = .10, η_p_^2^ = .10], task*diagnosis interaction [F(1,26) = 0.55, p = .47, η_p_^2^ = .02]. Group average values for the percentage of time spent fixating in each ROI can be seen in Table 5. The level of autistic traits that participants displayed also did not significantly predict changes in the duration of fixation within any ROI between conditions (see Table 6).

**Table 5:** Percentage duration of fixation in each ROI; Mean (SD) values

	ASD	TD
Head ROI		
Implicit mentalizing-action	4.64 (9.68)	2.32 (12.01)
Implicit either-action	4.32 (8.00)	0.37 (11.08)
Explicit mentalizing-non-mentalizing^a^	0.06 (18.05)	1.91 (13.13)
Poker chip ROI		
Implicit mentalizing-action	1.16 (6.15)	− 1.83 (8.76)
Implicit either-action	2.36 (5.58)	2.12 (11.96)
Explicit mentalizing-non-mentalizing^a^	4.12 (15.89)	0.75 (6.21)
Release point ROI		
Implicit mentalizing-action	− 5.34 (3.98)	− 1.99 (5.52)
Implicit either-action	− 6.04 (4.24)	− 3.67 (4.76)
Explicit mentalizing-non-mentalizing^a^	− 3.53 (6.81)	− 3.82 (2.70)

^a^Median (IQR) values presented as non-parametric tests were used

**Table 6:** Results of the linear regression analyses investigating relationships between the eye-tracking data and the level of autistic traits displayed

	Mentalizing-Action					Mentalizing-Either				
	*B*	*SE B*	β	*t*	*p*	*B*	*SE B*	β	*t*	*p*
Head ROI	− .72	2.05	− .07	− .35	.73	.26	1.86	.03	.14	.89
Poker Chip ROI	2.68	1.36	.36	1.98	.06	1.31	1.76	.14	.74	.46
Release Point ROI	− 1.42	.92	− .29	− 1.54	.14	− .99	0.85	− .22	− 1.17	.26

### Experiment 2

Participants in the ASD group displayed significant explicit mentalizing deficits (Median = − 1.88; IQR = 3.50) compared to matched controls (Median = − .74; IQR = 1.13; U = 61.50, p = .03, r = .39). The participant in the ASD group with the highest level of autistic traits was identified as an outlier in the linear regression analysis for the explicit task (Cook’s distance > 1 and leverage value > n/4), therefore this participant was removed from the linear regression analysis.

**Fig. 4:**
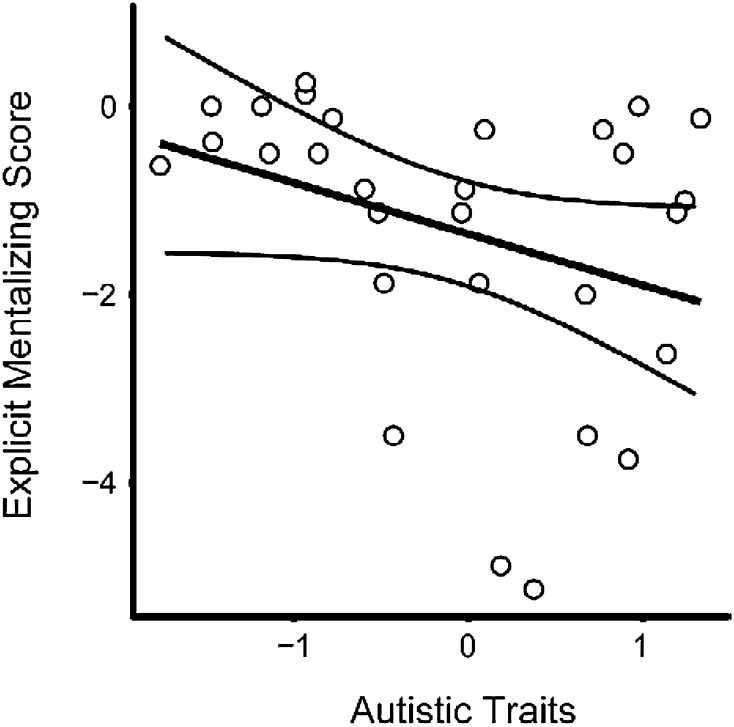
The relationship between the levels of autistic traits displayed and performances on the explicit mentalizing task. Although there was a trend of poorer explicit mentalizing performances with high levels of autistic traits, linear regression analysis found that the level of autistic traits displayed was not a significant predictor of performance F(1,27) = 3.42, p = .08, R^2^ = 0.11. The curved lines represent 95% confidence intervals

Participants with higher levels of autistic traits displayed poorer performances on the explicit mentalizing condition but this was a non-significant trend (mentalizing-non-mentalizing scores; F(1,27) = 3.42, p = .08, R^2^ = 0.11, 95% CI [− 1.15, 0.06] see Fig. 4).

Participants with ASD displayed typical changes in the duration of fixation between mentalizing and non-mentalizing conditions for all ROIs (Head ROI: U = 75.00, p = .32, r = .19: Poker chip ROI: U = 77.00, p = .36, r = .17: Release point ROI: U = 74.00, p = .29, r = .20). The level of autistic traits that participants displayed did not significantly predict changes in the duration of fixation between mentalizing and and non-mentalizing conditions within any ROI (Head ROI: F(1,26) = 2.23, p = .15, R^2^ = 0.08, 95% CI [− 10.77, 1.71]; poker chips ROI: F(1,26) = 1.63, p = .21, R^2^ = 0.06, 95% CI [− 2.04, 8.76]; release point ROI: F(1,26) = 1.63, p = .90, R^2^ < 0.001, 95% CI [− 1.65, 1.47]).

## Discussion

This study aimed to investigate the abilities of adults with ASD to both implicitly and explicitly infer the intentions of others. In the first experiment, participants completed a task where mentalizing was implicit. Here participants were asked to make decisions about who they wanted to interact with between pairs of actors and in some cases these social decisions required the intentions of the actors to be inferred in order to make the appropriate choice. In contrast, during the second experiment, participants were explicitly asked to report the intentions of actors. Our results showed that adults with ASD displayed explicit mentalizing deficits compared to matched controls. Adults with ASD did not display significant implicit mentalizing abilities. Furthermore, ASD participants did not display atypical fixation patterns during both the explicit and implicit experiments. Therefore, the explicit mentalizing deficits exhibited by adults with ASD cannot be explained by differences in fixation.

The explicit mentalizing deficit found with adults with ASD in this study supports a number of previous studies which found adults with ASD were impaired at explicitly inferring others’ intentions (Happé 1994; Kana et al. 2014; Moran et al. 2011). Our data are also consistent with reported difficulties for adults with ASD in everyday life (O’Neal 2013; The National Autistic Society 2014). However, some previous studies have not found a connection between ASD and impairments in explicitly inferring the intentions of others (McAleer et al. 2011; Ponnet et al. 2004; Roeyers et al. 2001; Schuwerk et al. 2014). This may be due to the simplicity of the stimuli used in these studies, e.g. passages of text and still images (Ponnet et al. 2004; Roeyers et al. 2001; Schuwerk et al. 2014). In contrast, our study used a task with complex, naturalistic stimuli more akin to social environments in which individuals are required to make judgements. The use of more simplistic stimuli in previous studies may have allowed some adults with ASD to explicitly infer the intentions of others, perhaps with the help of learned strategies, which are of less help in more complex and natural settings. In support of this argument, two previous studies have investigated the ability of adults with Pervasive Development Disorders (PDDs; including ASD) to infer mental states both using simple stimuli and complex, naturalistic stimuli (Ponnet et al. 2004; Roeyers et al. 2001). Their results showed that adults with PDDs were only impaired when complex stimuli were used.

In addition to the group analysis, we also investigated the relationship between autistic traits and performance across all participants. This additional analysis was conducted as ASD is a spectrum disorder rather than a dichotomous classification and our results clearly show that participants displayed a range of autistic traits (see Figs. 3, 4). The linear regression analysis showed that across all participants the wide range of autistic traits shown was negatively associated with performance on both implicit and explicit mentalizing tasks, but these remained non-significant trends. A previous study found no relationship between autistic traits and both explicit and implicit mentalizing abilities (Nijhof et al. 2016). However, this study did not recruit adults with an ASD diagnosis and therefore may have not had the range of autistic traits required to find a relationship between autistic traits and mentalizing performance.

Although a trend was found in our study for poorer implicit mentalizing abilities associated with higher levels of autistic traits, there was not a significant group difference in performance between those with ASD and their matched controls. This lack of clear evidence for a significant implicit mentalizing deficit in adults with ASD was unexpected. We had more participants in this study than in the explicit study, which revealed clear significant results, so it is unlikely the null result is simply due to insufficient statistical power. It is possible that if the data had been normally distributed, therefore allowing parametric analyses to be carried out, the interaction between task and participant group would not have been significant, reflecting comparable deficits on both tasks. However, the effect size was much larger for the explicit experiment (r = .39) compared to the implicit experiment (r = .19), supporting the presence of a significant explicit deficit but no clear implicit mentalizing deficit in these adults with ASD. The existing literature shows consistent implicit mentalizing deficits in adults with ASD (Castelli et al. 2002; Rosenblau et al. 2015; Schuwerk et al. 2014; Senju et al. 2009). The methods we used to measure mentalizing abilities may have contributed to the discrepancy between our findings and the previous literature. Our study measured implicit mentalizing abilities using a measurable behavioural outcome and performances were assessed relative to a control task. Previous implicit mentalizing studies in adults with ASD that used complex stimuli have either used eye-tracking data alone as a measure of mentalizing abilities (Schuwerk et al. 2014; Senju et al. 2009) or not included a control task (Rosenblau et al. 2015). Without the inclusion of a control task, it cannot be determined whether poorer performances linked to ASD are mentalizing-specific or more generalised deficits. Additionally, this study was the first to investigate abilities to implicitly infer intentions in adults with ASD; in contrast previous implicit mentalizing studies in adults have assessed abilities to infer others’ mental states and false beliefs (Castelli et al. 2002; Rosenblau et al. 2015; Schuwerk et al. 2014; Senju et al. 2009). Neuroimaging studies have shown that different brain areas are active during different types of mentalizing tasks (Gobbini et al. 2007; Pineda and Hecht 2009; Saxe and Powell 2006; Schurz et al. 2014), suggesting that the systems used depend on the specific mentalizing task being performed. Therefore, it is possible that ASD is related to more pronounced deficits on certain subcomponents of mentalizing than others.

The lack of clear evidence for a significant implicit mentalizing deficit in adults with ASD in the current study may also be due to the use of action stimuli; implicitly inferring others’ intentions from their actions may involve different processes than implicit mentalizing in the absence of action information. Actions with different intentions have been shown to display different kinematic profiles (Manera et al. 2011; Sartori et al. 2011). The dual-process model suggests that when intentions are inferred from others’ actions, these differences in action kinematics allow automatic, subconscious processing of intentional information in the observer’s own motor system before intentions are actively interpreted in a higher-level cortical system (de Lange et al. 2008; Keysers and Gazzola 2007; Spunt and Lieberman 2012; Uddin et al. 2007). Neuroimaging data suggest that in the absence of action information, others’ intentions aren’t subconsciously processed in the motor system (see a review and meta-analysis; Gallagher et al. 2000; Schurz et al. 2014). Therefore, because intentional information in our study was provided by differences in action kinematics, it is possible that subconscious processing of intentional information in the motor system allowed adults with ASD to select preferable kinematic profiles (required in our implicit task). Whereas, if intentional information was provided by other cues, not solely by differences in action kinematics, then a significant implicit mentalizing deficit may have been found. A larger number of implicit mentalizing studies have been carried out in young children than adults and a number of studies have shown that children with ASD can implicitly infer others’ intentions when intention is portrayed using action (Aldridge et al. 2000; Berger and Ingersoll 2014; Carpenter et al. 2001; Colombi et al. 2009; Liebal et al. 2008; Schietecatte et al. 2012) but not when intentions are portrayed by social-emotional cues such as eye gaze or facial expression (Berger and Ingersoll 2014; Vivanti et al. 2016). These data support the theory that inferring intentions from action kinematics involves different processes than inferring intentions using different cues and that implicitly inferring intentions from action kinematics is not significantly impaired in ASD.

Individuals with ASD and high levels of autistic traits also showed relatively poor performance on Either trials. It seems likely that mentalizing may have influenced the social judgments participants made during the Either condition even though, in principal, mentalizing was not required. The Either condition was included in this study in attempt to control for differences in the success of actions across mentalizing and non-mentalizing (Action) conditions. In the Mentalizing condition, unsuccessful actions were always seen and in the Action condition only successful actions were seen. The Either condition showed unsuccessful actions but did not require mentalizing in order to complete the task, if participants made their decisions based purely on the number of poker chips involved in the hand actions then they would make correct choices. However, previous evidence suggests that the intentionality of observed hand actions is automatically processed (Liepelt et al. 2008), and given participants were blind to the condition, from the participant’s perspective, the relevant feature of the action (number of chips/intention of the actor) only became clear after the second movie had been viewed. Thus, it may have been an effective strategy to pay attention to the intention of the actor in all trials. This may have affected performance in several ways. First, participants with higher levels of autistic traits may have wrongly attributed negative intent to the preferable actions (the actor attempting to pass more poker chips) in the Either condition resulting in incorrect choices. Second, reading actor intentions may have distracted participants from focussing on the number of chips being passed and thus the inclusion of both actor intentions and differential number of chips, may have placed a higher cognitive load on participants, compared to other conditions and this may have contributed to the relatively poor performances in this condition.

Despite the poorer explicit mentalizing abilities found in adults with ASD compared to matched controls in our study, fixation patterns were not different in the ASD group. The typical fixation patterns exhibited by adults with ASD in this study may also be due to the use of action stimuli. The majority of the literature reporting atypical fixation patterns in adults with ASD have found atypical fixation patterns during face processing, in particular, showing reduced fixation on the eyes (Dalton et al. 2005; Klin et al. 2002; Pelphrey et al. 2002; Sterling et al. 2008). In the current study, the actors’ faces were not shown and intentional information was portrayed by the kinematics of the actions alone. Adults with ASD may alter their eye movements appropriately according to differences in the mentalizing demand of the task when intentional information is portrayed by action kinematics but not when internal state inferences require face processing. This theory is supported by data from a previous study that showed that when adults with ASD naturalistically viewed videos and pictures of social scenes they displayed reduced fixation on people’s faces but showed equivalent fixation on bodies to control participants (Rigby et al. 2016). The typical eye-tracking data in conjunction with the explicit mentalizing deficit in the current study suggest that despite receiving the visual cues they needed, adults with ASD could not accurately interpret the social cues embedded within the action kinematics in order to explicitly infer the actors’ intentions. This dissociation between the behavioural data and the eye-tracking data has implications for future research assessing mentalizing abilities. Some previous studies have investigated mentalizing abilities using eye-tracking data alone (Schuwerk et al. 2014; Senju et al. 2009). However, our results demonstrate that poorer mentalizing abilities associated with high levels of autistic traits are not always accompanied by atypical visual fixation patterns. This is supported by previous research which showed that although adults with ASD spent less time fixating on the eyes of others, these atypical fixation patterns did not correlate with poorer mentalizing performances (Cassidy et al. 2013).

In conclusion, we found that adults with ASD were significantly impaired at explicitly but not implicitly inferring the intentions of others from their hand actions. Although there was a trend for adults with high levels of autistic traits to display poorer implicit mentalizing performances, this relationship did not reach significance. The lack of a significant implicit mentalizing deficit may be due to subconscious processing of intentional information when intentions are portrayed by action kinematics. Adults with ASD displayed typical fixation patterns when both implicitly and explicitly inferring the intentions of others. The inconsistency we observed between impaired explicit mentalizing but typical fixation patterns suggests that reduced abilities to explicitly infer intentions from hand actions cannot be attributed to dissimilarities in fixation patterns. Our findings suggest that future research should consider the stimuli used and assess mentalizing abilities with both behavioural and eye-tracking techniques.

## Electronic supplementary material

Below is the link to the electronic supplementary material.

Supplementary material 1 (WMV 1942 KB)

Supplementary material 2 (WMV 2840 KB)

Supplementary material 3 (WMV 2106 KB)
